# Calcium Bioavailability in the Soluble and Insoluble Fibers Extracted from *Opuntia ficus indica* at Different Maturity Stages in Growing Rats

**DOI:** 10.3390/nu12113250

**Published:** 2020-10-23

**Authors:** Monsserrat Mendoza-Ávila, Elsa Gutiérrez-Cortez, Michelle Quintero-García, Alicia Del Real, Eric M. Rivera-Muñoz, César Ibarra-Alvarado, Efraín Rubio, Daniel Jiménez-Mendoza, Isela Rojas-Molina

**Affiliations:** 1 Programa de Maestría en Ciencias de la Nutrición Humana, Facultad de Ciencias Naturales, Universidad Autónoma de Querétaro, Av. de las Ciencias S/N, Juriquilla C.P. 76230, Querétaro, Mexico; monsse_ph@hotmail.com; 2 Laboratorio de Química Medicinal, Facultad de Química, Universidad Autónoma de Querétaro, Cerro de las Campanas S/N, Querétaro C.P. 76010, Mexico; adr_mich@hotmail.com (M.Q.-G.); cibarra@uaq.mx (C.I.-A.); 3 Laboratorio de Procesos de Transformación y Tecnologías Emergentes en Alimentos, Facultad de Estudios Superiores-Cuautitlán, Universidad Nacional Autónoma de Mexico, Km 2.5 Carretera Cuautitlán–Teoloyucan, San Sebastián Xhala, Cuautitlán Izcalli C.P. 54714, Mexico; elsaneqpm@yahoo.com.mx; 4 Programa de Maestría en Ciencias Químico Biológicas, Facultad de Química, Universidad Autónoma de Querétaro, Cerro de las Campanas S/N, Querétaro C.P. 76010, Mexico; 5 Centro de Física Aplicada y Tecnología Avanzada, Universidad Nacional Autónoma de Mexico, Jurqiuilla C.P. 7600, Querétaro, Mexico; adelreal@unam.mx (A.D.R.); erivera@unam.mx (E.M.R.-M.); 6 Centro Universitario de Vinculación y Transferencia de Tecnología, Benemérita Universidad Autónoma de Puebla, Centro Universitario, Col. San Manuel S/N, Puebla C.P. 72540, Mexico; efrainrubio@yahoo.com; 7 Departamento de Ingeniería Física, División de Ciencias e Ingenierías, Universidad de Guanajuato, Campus León, Lomas del Bosque 103, Col. Lomas del Campestre, León C.P. 37150, Guanajuato, Mexico; jmd_pepe@hotmail.com; 8 Departamento de Ingeniería Electromecánica, Tecnológico Nacional de México/ITS de Purísima del Rincón. Blvd. Del Valle 2301, Col. Guardarrayas, Purísima del Rincón, Purísima del Rincón Guanajuato C.P. 36413, Mexico

**Keywords:** calcium bioavailability, soluble dietary fiber, insoluble dietary fiber, *Opuntia ficus indica*, childhood, adolescence

## Abstract

Childhood and adolescence are crucial stages of life for bone health. Therefore, an adequate calcium intake and a healthy life style constitute the main strategies to prevent the risk of osteoporosis-related fractures during adulthood. It has been demonstrated that inclusion of indigestible carbohydrates in foods can help improve calcium absorption in growing stages. The objective of this study was to evaluate the effect of supplementation of soluble and insoluble fibers extracted from *O. ficus indica* cladodes on calcium bioavailability. Male Wistar rats 4-week old were fed diets added with soluble and insoluble fibers extracted from *O. ficus indica* cladodes at early and late maturity stages, as the only source of calcium. The mineral content, bone mineral density (BMD), physical, microstructural, and biomechanical properties of rat femurs were determined. The bones of rats fed with diets containing a soluble fiber extracted from *O. ficus indica* at early and late maturity stages exhibited better bone properties (resistance to fracture, microarchitecture, and calcium content) than control rats and rats fed with an insoluble fiber from *O. ficus* *indica* cladodes at both maturity stages. As expected, based on these results, the BMD values were higher in adolescent and pubertal rats fed with a diet containing the *O. ficus indica* soluble fiber. These results demonstrate that the soluble fiber from *O. ficus indica* cladodes is indeed a valuable source of bioavailable calcium, which contributes to improve physical, densitometric, biomechanical, and microstructural properties of bone in growing rats.

## 1. Introduction

The human body in the adult stage contains about 1.2 kg of calcium and 99% of this mineral is found in mineralized tissues such as bones and teeth. The remaining 1% of calcium is located in blood, muscles, and other tissues, where it participates in vascular contraction/vasodilation, muscle contraction, nerve transmission, glandular secretion, etc. [1,2]. Peak bone mass (defined as the amount of skeletal tissue and strength achieved at the end of the growth period) is an important factor that determines the risk of osteoporotic fractures happening in adulthood [3]. Calcium intake and physical activity are recognized strategies to promote maximal bone health from childhood through young adulthood [4]. The recommended calcium intake for adolescents varies from 1000 to 1300 mg/day depending on sex and age [5,6].

On the other hand, it has been reported that prebiotics such as inulin-type fructans, fructooligosaccharides, galactooligosaccharides, sugar alcohols, complex polysaccharides, and several non-digestible substrates are fermented in the large intestine by microbiota. This increases mineral absorption, promoting bone mineralization and improving structural and mechanical properties of bone in ovariectomized rats and young healthy humans [7,8]. Hence, it is important to investigate the effect of novel dietary fibers included in traditional foods of some populations on bone health.

The Mexican traditional diet comprises certain calcium-rich foods, such as *Opuntia ficus indica* cladodes, which are consumed as vegetables at an early maturity stage (nopalitos) [9]. *O. ficus indica* mucilage is a hydrocolloid composed of highly branched polysaccharides, which contains 10% uronic acids, arabinose, galactose, rhamnose, and xylose. The backbone of such polysaccharides consists of α-dgalacturonic acid units linked 1–2 to β-lrhamnose units linked 1–4 with branches attached to the C-4 position. Branches being integrated of galactose oligosaccharides, which carry l-arabinose and d-xylose as substituents [10]. The soluble dietary fiber content of *O. ficus indica* (constituted mainly by mucilage) is higher in cladodes at an early maturity stage than in cladodes at a late maturity stage [11]. Moreover, the soluble dietary fiber from *O. ficus indica* contains calcium salts, predominantly calcium carbonate, which is potentially bioavailable. By contrast, in the insoluble dietary fiber, calcium is primarily found as calcium oxalate, in which bioavailability is significantly lower [12,13].

Recently, our research group demonstrated that calcium from *O. ficus indica* cladodes at a late maturity stage is more bioavailable than calcium found in cladodes at an early maturity stage [14]. We also found evidence indicating that calcium in the soluble fiber of *O. ficus indica* cladodes at late maturity stage was bioavailable in an ovariectomized rat model of postmenopausal bone loss [15]. Considering that our previous findings showed that *O. ficus indica* cladodes represent a good source of bioavailable calcium, we hypothesized that regardless of the maturity stage of *O. ficus indica* cladodes, the calcium bioavailability in the soluble dietary fiber (mucilage) will be significantly enhanced compared to that in the insoluble dietary fiber of this cactus.

The present study was aimed to further investigate the bioavailability of calcium contained in the soluble and insoluble dietary fibers of *O. ficus indica* cladodes (at early and late maturity stages) and evaluate their effect on bone quality parameters in growing rats. 

## 2. Materials and Methods 

### 2.1. Vegetal Material

*Opuntia ficus indica* cladodes were cultivated with an organic fertilizer during the spring of 2016 in the experimental field of the Engineering Department of the Autonomous University of Querétaro. Cladodes were collected at two maturity stages: (1) Early stage (cladodes of 25 to 60 days after sprouting) and (2) late stage (cladodes of 100 to 135 days after sprouting). The extraction of the soluble and insoluble fibers from cladodes was conducted according to the methodology reported by Rojas-Molina et al. [12].

### 2.2. Determination of Mineral (Calcium, Phosphorus, Magnesium, and Potassium) Contents in the Soluble and Insoluble Dietary Fiber of O. ficus indica Cladodes

The content of calcium (Ca), phosphorus (P), magnesium (Mg), and potassium (K) in the soluble and insoluble dietary fiber of *O. ficus indica* cladodes was quantified by inductively coupled plasma mass spectrometry (ICP-MS) using an ICP-EOS Variant 730-ES (Santa Clara, CA, USA) equipment following the official methods, as previously reported [16,17].

### 2.3. Experimental Diets

The experimental diets were prepared either with the soluble dietary fiber of *O. ficus indica* (at early and late maturity stages) or the insoluble dietary fiber. The diets were based on the formulation of the American Institute of Nutrition Rodent Diets for growing rats (AIN-93G) [18] with some modifications, which included the addition of a vitamin mix (AIN-93-VX, Harlan Inc. IN, USA, TD 94047) and a mineral mix without calcium (AIN-93-MX, Harlan Inc. IN, USA, TD 04374) (Table 1). The calcium content in all the diets was 5 g/kg diet. In the case of the control diets the source of calcium was calcium carbonate (Merck 2066, Darmstadt, Germany). Furthermore, different amounts of dehydrated soluble or insoluble dietary fiber of *O. ficus indica* at different maturity stages were added to the experimental diets as the only source of calcium to achieve the required calcium content [14]. Energy values were calculated with standard factors such as 4 kcal for available carbohydrates and proteins and 9 kcal for lipids. 

### 2.4. Experimental Design

Thirty-five 4-week-old male Wistar rats were obtained from the Animal Production Unit of the Neurobiology Institute of the National Autonomous University of Mexico, and were housed separately. After a week of adaptation, the animals were separately housed in metabolic cages and kept in a temperature (22 ± 2 °C) and light-controlled (12-h day–night cycle) room. The experimental rats were randomly classified into five experimental groups with seven rats in each one, as follows: (1) The control group fed with a diet AIN-93M for the maintenance of growing rodents, (2) the group fed with a soluble dietary fiber of *O. ficus indica* at an early maturity stage (FS-TM), (3) the group fed with a soluble dietary fiber of *O. ficus indica* at a late maturity stage (FS-TD), (4) the group fed with an insoluble dietary fiber of *O. ficus indica* at an early maturity stage (FI-TM), and (5) the group fed with an insoluble dietary fiber of *O. ficus indica* at a late maturity stage (FI-TD). The study was approved by the Bioethics Committee of the Natural Sciences Department of the Autonomous University of Queretaro. The animals were fed with experimental diets and deionized water *ad libitum* for 6 weeks. The food intake was recorded daily and the body weights were measured every week. Additionally, the food efficiency was determined by the relationship between weight gain and food intake at the end of the experiment [19].

### 2.5. Assessment of Femoral Dimensions and Weight

Once the experimental period was concluded, the rats were sacrificed by decapitation after fasting for 12 h. The femurs of each rat were removed and weighed, while the length, width, and thickness of the bones were measured using a Vernier (Absolute Digimatic, Mitutoyo, Japan). Afterwards, the bones were stored at −20 °C for further analysis [14].

### 2.6. Analysis of Biomechanical Properties of Rat Femurs

The biomechanical properties of the femoral bones were analyzed through the force required to break the bones by using a material testing machine (Zwick/Roell Ulm Germany, Mod. Z005, load cell 5000N), using the TestXpert Intelligent testing version 12.0 software [14].

### 2.7. Determination of Mineral Content in Rat Femoral Bones

The Ca, P, K, and Mg contents in femurs were determined by ICP-MS according to Hernández-Becerra et al. (2017) [14].

### 2.8. Analysis of Microstructural Properties of Rat Femoral Bones

The trabecular separation (Tb.Sp), trabecular thickness (Tb.Th), and cortical thickness (Ct.Wi) in femoral bones were determined by scanning electron microscopy (Jeol JSM 6060LV, Tokyo, Japan), as previously described [14,20]. Briefly, the regions of interest for bone quality assessment were selected as follows: Ct.Wi was measured in the femoral cortical bone at the level of the distal metaphysis; Tb.Sp and Tb.Th were measured in the proximal epiphysis near the epiphyseal growth plate within the same microscope field. For each experimental group, 20 measurements were taken from seven different rats and the averages were reported.

### 2.9. Assessment of Bone Mineral Density

The experimental animals were subjected to a densitometry at the beginning and at the end of the experiment by using a simple X-ray equipment (Satelec X-mind^®^ Mérignac, France) with a 70 kV, a current of 8 mA potential, and a 0.177 × 10^−10^ m wavelength (Toshiba X-ray tube DG-073B-DC Tokyo, Japan) [14,17].

### 2.10. X-ray Diffraction Analysis of Femoral Bones

The X-ray diffraction patterns were obtained by using a Rigaku Ultima IV diffractometer (Tokyo, Japan) according to the method as reported by Hernández-Becerra et al. (2020) [17]. The crystallite size (CS) was obtained with the Scherrer’s equation (Equation (1)) [21].

(1) $$CS=\frac{k\lambda }{\beta Cos\theta }$$

In addition, the absolute degree of crystallinity (ADC) was obtained from X-ray diffraction data by measuring the ratio of the crystalline area and the total area in the diffractogram [21] (Equation (2)).

(2) $$ADC\;\left(\%\right)=\frac{Total\;area-amorphous\;area}{Total\;area}\times \;100\;\;\;\;\;\;$$

### 2.11. Statistical Analyses

All of the values are expressed as the mean ± standard deviation of the mean (SD). All data were analyzed using a one-way analysis of variance (ANOVA) followed by Tukey’s test with α = 0.05 and using the GraphPad Prism 6 procedure (GraphPad Software, Inc., San Diego, CA, USA). The calcium source within the diets was considered to be the variation factor. At least three to seven replicates were carried out in each experiment, depending on the response factor (the evaluated parameter).

## 3. Results

### 3.1. Mineral Content in the Soluble and Insoluble Fiber Extracted from O. ficus indica

The average Ca, P, K, and Mg contents in the soluble and insoluble dietary fiber extracted from *O. ficus indica* cladodes at early and late maturity stages are shown in Table 2. Statistically significant differences (*p* ≤ 0.05) were found in Ca, P, K, and Mg contents between the samples. 

The Ca content in the soluble and insoluble fibers of the cladodes at late maturity stage (FS-TD and FI-TD) was significantly higher (*p* ≤ 0.05) than that in the soluble and insoluble dietary fiber of the cladodes at the early maturity stage (FS-TM and FI-TM). 

The P content in the FS-TM was significantly lower (*p* ≤ 0.05) than the one of the FS-TD. By contrast, the content of this mineral in the FI-TM was higher (*p* ≤ 0.05) than the one observed in the FI-TD. Nevertheless, the addition of the P content in the fiber of cladodes at the early maturity stage (410 mg/100 g) was higher than the one detected in the fiber of cladodes at late maturity stage (380 mg/100 g). A similar trend was observed for the case of K content, which was significantly higher (*p* ≤ 0.05) in FS-TM and FI-TM (4000 mg/100 g) than that of FS-TD and FI-TD (3340 mg/100 g). At the other extreme, FS-TD and FI-TM showed higher levels of Mg than FS-TM and FI-TD. 

### 3.2. Body Weight Gain, Food Intake, and Food Efficiency in Rats Fed Control and Experimental Diets

Table 3 shows weight gain, food intake, and food efficiency in experimental rats. There were no statistically significant differences (*p* ≤ 0.05) in these parameters, which indicated that the consumption of the soluble and insoluble dietary fiber of *O. ficus indica* cladodes does not affect weight gain, food intake, and food efficiency.

### 3.3. Assessment of Dimensions and Biomechanical Properties of the Femurs of the Rats Fed the Soluble and Insoluble Dietary Fiber of O. ficus indica as a Calcium Source

The dimensions and biomechanical properties of the femurs of rats fed with the experimental diets are shown in Table 4. No significant differences (*p* ≤ 0.05) were detected in the mean values of length of the rat’s femoral bones. The weight of the femurs of the rats fed the FS-TD was significantly higher (*p* ≤ 0.05) than that of rats fed the FS-TM, FI-TM, and FI-TD. Moreover, the width and thickness of the femoral bones in rats fed the FS-TD showed the highest values.

Regarding biomechanical testing of bones, the femurs of rats fed with FS-TM and FS-TD exhibited the highest values in the compression test (671.4 ± 10.8 and 633.10 ± 13.8 N, respectively), which were even higher than those observed for the control group (614.74 ± 10.6). While the femurs of rats fed with the FI-TM and FI-TD presented the lowest values with statistically significant differences (*p* ≤ 0.05). The results obtained from the flexion test showed a behavior similar to those of the compression test: The femurs of the rats fed the FS-TM and FS-TD exhibited the highest values (102.3 ± 1.9 and 109.7 ± 9.0 N, respectively), which were significantly higher (*p* ≤ 0.05) than those of the control group (87.8 ± 4.3). Whereas, the bones of rats fed the FI-TM and FI-TD showed the lowest compression values. Concerning the Youngs modulus (E), the femurs of rats fed the FS-TD, FS-TM, and FI-TM exhibited significantly higher (*p* ≤ 0.05) E values than the ones of the control group (236.9, 70.5, and 45.43%, respectively) (Table 4).

### 3.4. Assessment of Bone Mineral Contents 

The mineral contents (Ca, P, Mg, and K) of the rat femoral bones are shown in Table 5. Significant differences (*p* ≤ 0.05) were observed between the Ca content of the bones from rats of different experimental groups. The bones from rats fed the FS-TD group exhibited the highest calcium content (447.7 ± 2.7 mg/g), which was even higher than the one of bones of rats of the control group (385 ± 5.7 mg/g), which represents a 16.3% calcium content increase. The femurs of the rats fed with FI-TD showed the lowest Ca content (218 ± 3.5 mg/g). Regarding P, the highest content of this mineral was found within the bones of rats fed with the FS-TD and rats of the control group with values of 91.4 ± 1.0 and 92.8 ± 1.1 mg/g, respectively, which did not demonstrate any significant difference (*p* ≤ 0.05). The P content of the femurs of rats fed with FI-TM was the lowest amongst all tested groups. No significant differences were observed in the Mg content of the femurs from rats fed with the FS-TD and FI-TD (1.78 ± 0.01 and 1.87 ± 0.1 mg/g, respectively) and control rats (1.88 ± 0.01 mg/g), while the bones of rats fed with FS-TM and FI-TM showed the lowest values.

Statistically, significant differences (*p* ≤ 0.05) were observed between the K content of the femurs pertaining to rats of the experimental groups. Nonetheless, the highest content of this mineral was found in the femurs of rats fed with FS-TD and FI-TM (0.229 ± 0.002 and 0.193 ± 0.001 mg/g, respectively), it was lower than the K content of the control rats (0.378 ± 0.003 mg/g).

Regarding the Ca/P molar ratio, the bones from rats fed the FS-TD presented the highest value (3.75 ± 0.183 mg/g), which was 15.6% higher than the ratio of the femoral bones from rats of the control group (3.20 ± 0.009 mg/g), which did not demonstrate a significant difference (*p* ≤ 0.05) against the Ca/P molar ratio of the bones from rats fed with the FS-TM. The lowest values were observed in the femurs of rats fed with FI-TM and FI-TD (2.20 ± 0.009 and 1.90 ± 0.013, respectively).

### 3.5. Assessment of Microstructural Properties of Femurs of Rats Fed the Soluble and Insoluble Dietary Fiber of O. ficus indica as the Only Calcium Source by SEM

**Figure 1:**
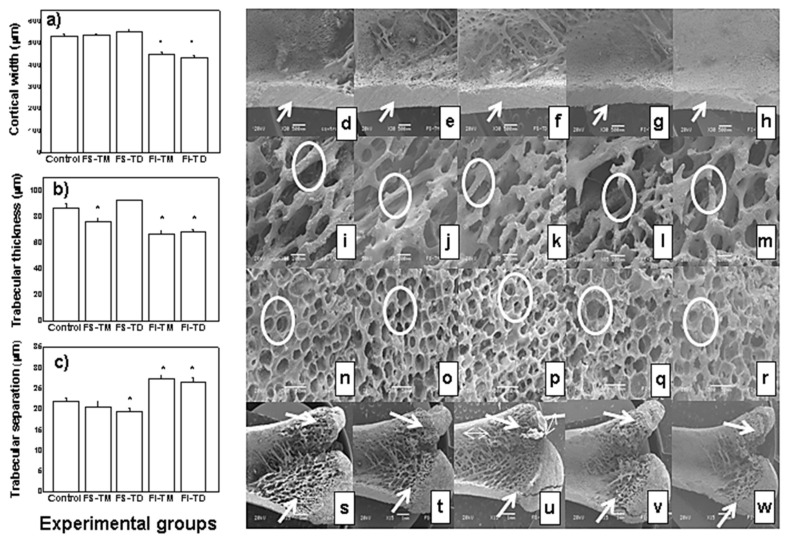
Microstructural parameters of the femurs of male Wistar growing rats fed with the control diet and the soluble and insoluble fiber of *O. ficus indica* cladodes at early and late maturity stages as the only dietary calcium source. (**a**) Indicates the cortical width of the femoral diaphysis (Ct.Wi); (**b**) displays the trabecular thickness of femoral metaphysis (Tb.Th); (**c**) exhibits trabecular separation of the femoral epiphyseal line growth (Tb.Sp). The values represent the mean ± SD *n* = 7. * Significantly different from the values of bones from rats fed the control diet (*p* ≤ 0.05). Scanning electron microscopy images left to right: Control, FS-TM, FS-TD, FI-TM, and FI-TD groups. The micrographs (**d**–**h**) show the cortical width of femoral diaphysis (35×). The micrographs (**i**–**m**) show the trabecular thickness in femoral bones (85×). The micrographs (**n**–**r**) show details of the trabecular tissue in femoral bones (500×). The micrographs (**s**–**w**) show the inner part of the longitudinally sectioned femur from the line between condyles towards the diaphysis (15×).

Figure 1 exhibits the structural parameters and micrographs obtained by SEM of epiphyses, metaphyses, and diaphyses of the femurs of rat femoral bones. The cortical width (Ct.Wi) of femoral diaphyses of control rats and rats fed with the FS-TM and the FS-TD displayed significantly higher values (*p* ≤ 0.05) than those of the bones of rats fed with FI-TM and FI-TD (Figure 1a), which is clearly shown in their respective micrographs (Figure 1d–f, respectively). Figure 1b demonstrates trabecular thickness (Tb.Th) of the rat femoral bones. Tb.Th was significantly greater (*p* ≤ 0.05) in femurs of the rats fed the FS-TD and the control rats (Figure 1i,j, respectively). It is noteworthy to mention that the trabeculae of the rats fed with FI-TM and FI-TD (Figure 1l,m, respectively) exhibited micro-cracks (see circles). Regarding trabecular separation (Tb.Sp) of the femoral epiphyseal line growth (Figure 1c), the rats fed with FI-TM and FI-TD showed significantly greater values (*p* ≤ 0.05) against those of the rats fed with FS-TM, the FS-TD, and the control rats (Figure 1c). The images in Figure 1n–p show that Tb.Sp within the bones of the control rats and rats fed with FS-TM and FS-TD is lower than that of bones from rats fed with FI-TM and FI-TD (see circles). Finally, micrographs of the bones of control rats (Figure 1s), rats fed with FS-TM (Figure 1t), and rats fed with FS-TD (Figure 1u) indicated that the trabecular tissue area extends to the diaphysis (see arrows); while this area is smaller in the bones of rats fed with FI-TM and FI-TD (Figure 1v,w). Furthermore, the greater trochanter of the femurs from the rats fed the FI-TM and the FI-TD is thinner and more prominent than the one of the bones from rats belonging to the other experimental groups (see arrows).

### 3.6. Assessment of Bone Mineral Density of Femoral Bones

**Figure 2:**
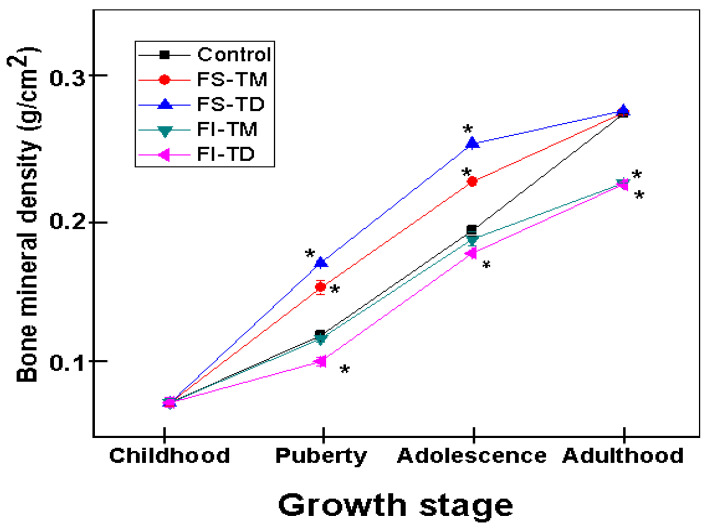
Bone mineral density (BMD) of the femurs of male Wistar growing rats fed with the soluble and insoluble fibers extracted from *O. ficus indica* cladodes at early and late maturity stages as the only dietary calcium source. The values represent mean ± SD *n* = 7. * Significantly different from the values of the bones from rats fed with the control diet at the same growing stage (*p* ≤ 0.05).

The bone mineral density (BMD) of experimental animals at different growth stages is shown in Figure 2. The control rats and rats fed the FS-TM and FS-TD exhibited the highest BMD values at all stages during the growing period. Consequently, in the adulthood stage, the BMD of rats belonging to these groups was significantly higher (*p* ≤ 0.05) than those of the rats fed the FI-TM and FI-TD.

### 3.7. Assessment of Crystallographic Analysis of Femoral Bone 

**Figure 3:**
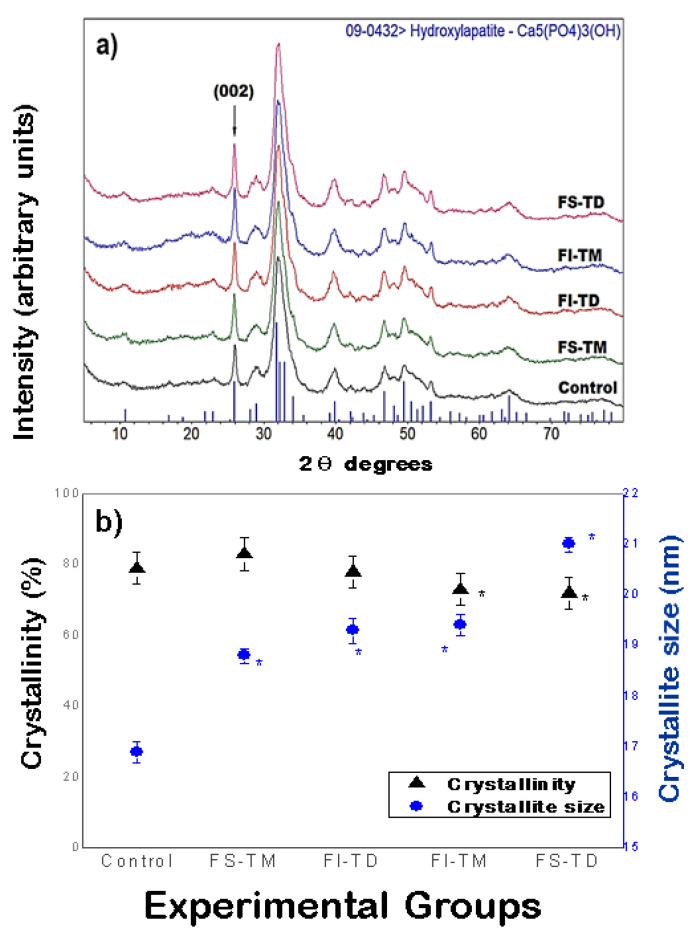
(**a**) X-ray diffractograms of the femurs of male Wistar growing rats fed with soluble and insoluble fiber of *O. ficus indica* cladodes at early and late maturity stages as the only dietary calcium source and control diet. Identification of the crystalline phase was carried out by using the ICDD-PDF file # 09-0432, corresponding to hydroxyapatite. (**b**) Crystallinity (%) and crystal size in femurs of the experimental groups. FS-TM: Soluble fiber from *O. ficus indica* at early maturity stage, FS-TD: Soluble fiber from *O. ficus indica* at late maturity stage, FI-TM: Insoluble fiber from *O. ficus indica* at early maturity stage, FI-TD: Insoluble fiber from *O. ficus indica* at late maturity stage. The values represent the mean ± SD *n* = 7; * significantly different from the values of the bones from rats fed with the control diet (*p* ≤ 0.05).

Figure 3a shows the X-ray diffraction patterns obtained from the femurs of rats fed the FS-TM, FI-TD, FI-TM, FS-TD, and control animals. In all patterns, a single crystalline phase is identified, which corresponds to hydroxyapatite (HAp), identified with the ICDD-PDF# 09-0432 file, in which Bragg reflections appear as vertical blue lines at the bottom of the figure. These results are in accordance with the composition of the mineral phase of the bone tissue, in which the main crystalline phase corresponds to HAp.

Crystallinity (%) and crystal size (nm) in femurs of the experimental groups are shown in Figure 3b. The crystal size was obtained by using the Scherrer equation (Equation (1)) applied to the (002) peak, located at 25.88 degrees in 2θ scale (indicated in the figure) and a λ = 0.15402 nm. The change in the percentage of crystallinity between the femurs of the rats fed the FS-TM and FS-TD was 11%, while the change between the femurs of the rats fed the FI-TD and FI-TM was 5%. Nonetheless, an 11.7% increase in crystal size between the bones of the rats fed the FS-TM and FS-TD was observed. Whereas, a variation in crystal size between femurs of the rats fed the FI-TD and FI-TM was only 0.05%. Interestingly, femurs of the group fed the FS-TM exhibited a significantly higher crystallinity value (*p* ≤ 0.05) than the one of the bones from the rest of the groups. The femoral bones of the rats fed with the FS-TD displayed a significantly larger crystal size (*p* ≤ 0.05). 

Table 6 shows the *r* values between BMD, mechanical, microstructural properties, mineral contents, and crystalline properties of femurs from rats of all experimental groups. Prior to these analyses, a normal distribution check was carried out on all data. Stronger correlations (*r* ≥ 0.5) were found between DMO (from adolescence to adulthood) and biomechanical properties (Fmax, Pmax, E), Ca/P molar ratio, and microstructural properties (Ct.Wi, Tb.Th, Tb.Sp), as well as between the Ca content in the femurs and microstructural properties (Ct.Wi, Tb.Th, Tb.Sp). Concerning crystalline properties of the femurs (crystallinity and crystal size), a strong correlation (*r =* 0.86) was observed between Young’s modulus (E) and crystal size. 

## 4. Discussion

Ca, K, and Mg contents in the soluble and insoluble fiber extracted from *O. ficus indica* cladodes coincide with previous studies [9,11,14,22,23], which indicate that *O. ficus indica* is an important source of minerals, mainly Ca and Mg. On the other hand, the biomechanical properties of femoral bones from rats fed the soluble fiber obtained from early and late maturity stage cladodes were better than those of the rats fed with the insoluble fiber, as demonstrated by significantly higher Fmax, Pmax, and E values. These results agree with previous findings that demonstrated that bone strength and resilience were improved in growing rats fed diets supplemented with soluble fibers [24,25]. It has been proposed that fiber, after being fermented, might improve Ca absorption through the production of specific: SCFA (i.e., butyrate, acetate, propionate) by promoting a reduction of pH, thereby increasing the solubility and transcellular absorption of mineral ions, such as Ca^2+^ [26]. Differences in the Ca content of femoral bones of rats belonging to the different experimental groups can be attributed to the differences in the crystalline structure of Ca found in the soluble and insoluble fiber of *O. ficus indica* cladodes [12]. The Ca increment in bones of the rats fed the soluble fiber of cladodes at a late maturity stage (FS-TD) demonstrates that this type of fiber is a source of bioavailable Ca, which is efficiently retained in bone tissues. Similar results were observed when growing rats were fed with fructooligosaccharides [27] and fructans [28]. There were no modifications detected in the Mg content of the femurs from the rats fed with soluble and insoluble fibers from *O. ficus indica* cladodes at any maturity stage. Concerning this, Lobo et al. [25] observed that Mg levels of tibial bones of rats fed with a diet rich in inulin-type fructans increased. The K content in femurs diminished in rats fed with t *O. ficus indica* fibers; this result differs from what we found in the bones of growing rats fed with *O. ficus indica* cladodes, which exhibited higher levels in the K content [14]. The differences found between the results reported previously and our data could be explained by considering differences in the length of the feeding period in the food matrix [27,29]. The P content diminished in rats fed with the insoluble fiber of *O. ficus indica* cladodes at an early and late maturity stage. This same behavior was observed in rats fed with the soluble fiber of cladodes at an early maturity stage. While the rats fed with the soluble fiber of cladodes at a late maturity stage did not show any difference in the femoral P level with respect to that of the control rats. These results agree with what we previously demonstrated in bones of rats fed *O. ficus indica* cladodes [14]. As expected, based on differences found in the Ca and P contents of the rat femoral bones, the Ca/P ratio varied within the different experimental groups, which showed Ca/P ratios ranging from 1.9 to 3.7. These results confirm what we previously found in rats fed with the *O. ficus indica* cladodes, in which the femoral Ca/P ratio was directly associated to the bone mineral density (BMD) [14]. In regards to such findings, a direct relationship between the loss of bone mass and a low Ca/P ratio has been observed [30]. Accordingly, in this study, we found a high correlation (*p* < 0.001) between the femoral Ca/P ratio values and the BMD in adolescent and adult rats (*r* = 0.856 and 0.946, respectively, see Table 6). Concerning the microstructural properties, the femurs of the rats fed with the soluble fiber of cladodes at a late maturity stage, showed an improved trabecular separation (Tb.Sp), trabecular thickness (Tb.Th), and cortical thickness (Ct.Wi), as we previously demonstrated in growing rats fed with *O. ficus indica* cladodes at a late maturity stage [14]. The groups fed with the soluble fibers extracted from *O. ficus indica* cladodes, regardless of their maturity stage, showed the highest BMD values. These findings coincide with previous reports, which indicated that prebiotics are capable of improving the BMD of bones [24,28,31,32]. In all the experimental groups, the BMD gain was faster in the pubertal stage, as we previously observed in rats fed with the *O. ficus indica* cladodes at early and late maturity stages [14]. The rats fed with the insoluble fiber of *O. ficus indica* cladodes exhibited low BMD values, which can be attributed to the presence of calcium oxalates and low fermentable components (cellulose and lignin) in this type of fiber [12]. These results are consistent with our previous findings [15], in which we detected higher BMD values in ovariectomized rats fed with the soluble fiber from *O. ficus indica* than those observed in ovariectomized rats fed with the insoluble fiber. Moreover, ovariectomized rats fed with a diet containing the soluble fiber showed decreased serum levels of biomarkers related to bone remodeling (alkaline phosphatase, osteocalcin, and N-terminal propeptide of type I procollagen), which were consistent with improved calcium absorption. It is worth mentioning that the presence of micronutrients involved in the metabolic processes related to osseous remodeling, which were added to the experimental diets through the vitamin and mineral mixtures, as well as the soluble fiber of *O. ficus indica* that contains, vitamin D, vitamin K, magnesium, phosphorus, silicon, zinc, potassium, and boron [12,18,23,33], could have also contributed to an increment in bone mass formation. The X-ray diffraction analysis of rat femurs showed that: As the crystallinity decreased, the crystal size of HAp increased, which may be related to the amount of calcium atoms, since bones of rats fed with the soluble fiber of cladodes at a late maturity stage, which displayed the highest Ca/P ratio, also exhibited the largest HAp crystal size. The femoral Ca/P ratio of rats fed with the FS-TD (3.75) is greater than the stoichiometric ratio of HAp (1.67) [34], this implies that the amount of Ca atoms increased within the structure of HAp, since the calcium atomic size is greater than the one of the other constituent elements of HAp. It has been reported that the crystal size is determined by its elemental composition [17]. The benefits of soluble fibers extracted from *O. ficus indica* on bone health was supported by the strong correlations between BMD, biomechanical properties, microstructure (microarchitecture), and mineral content in the femur of experimental animals (see Table 6) as has been corroborated by other groups, that used similar experimental models [24,25,31,32,35].

## 5. Conclusions

In the present study, we found that femoral bones of growing rats fed with the soluble fiber obtained from *O. ficus indica* cladodes at the early and late maturity stage improved the calcium content, the bone’s mineral density, and the biomechanical and microstructural properties. These findings indicate that the soluble fiber extracted from *O. ficus indica* cladodes, regardless of their maturity stage, constitute a valuable source of bioavailable dietary calcium. Therefore, the consumption of this type of fiber might be a strategy to increase the calcium intake of Mexican children and adolescents. Further studies are needed to identify the mechanisms through which, the soluble fiber extracted from *O. ficus indica* helps improve the bone’s health during the growing stage of individuals.

## Figures and Tables

**Table 1:** Ingredient composition of the experimental diets containing soluble and insoluble dietary fiber extracted from *Opuntia ficus indica* cladodes as the only source of calcium (g/kg diet).

Ingredients	Control	FS-TM ^a^	FS-TD ^a^	FI-TM ^a^	FI-TD ^a^
Corn starch	629	609	604	609	609
Sucrose	100	100	100	100	100
Casein ^b^	200	190	195	191	196
Soybean oil	70	70	70	70	70
Fiber ^c^	50	42	48.3	35	38
MixMin ^d^	49	56	57	60	62
Mix Vit ^e^	10	10	10	10	10
L-Cystine	3	3	3	3	3
Choline bitartrate	2.5	2.5	2.5	2.5	2.5
CaCO_3_^f^	12.5	-	-	-	-
*O. ficus indica* dietary fiber ^g^	-	131	97	146	100

^a^ Diets prepared with: Soluble dietary fiber of *O. ficus indica* at an early maturity stage (FS-TM); soluble dietary fiber of *O. ficus indica* at a late maturity stage (FS-TD); insoluble dietary fiber of *O. ficus indica* at an early maturity stage (FI-TM); insoluble dietary fiber of *O. ficus indica* at a late maturity stage (FI-TD). ^b^ Casein Sigma Chemical, Inc. St. Louis MO, USA C-7078; ^c^ α-Cell Fiber Solft Zolca MPbiomedical, Santa Ana CA, USA. In diets prepared with the dietary fiber of *O. ficus indica*, the fiber content was adjusted to 50 g/kg diet, taking into consideration the fiber added to the diets; ^d^ mineral mix without calcium (AIN-93-MX,Harlan Inc. IN, USA, TD 04,374); ^e^ vitamin mix (AIN-93-VX, Harlan Inc. IN, USA, TD 94,047); ^f^ control diet containing CaCO_3_ (Merck 2066 Darmstadt, Germany) as calcium source; ^g^ in experimental diets, *O. ficus indica* dietary fiber powder provided 5 g/kg of calcium, as well as the carbohydrates, proteins, and lipids that complemented the nutritional requirements in the experimental diets (AIN-93G).

**Table 2:** Mineral contents in the soluble and insoluble dietary fiber extracted from *O. ficus indica* cladodes at different maturity stages (mg/100 g).

Fiber	Calcium	Phosphorus	Potassium	Magnesium
FS-TM	3620.0 ± 12.6	270.0 ± 17.1	1960.0 ± 18.6	370.0 ± 11.8 *
FS-TD	4870.0 ± 23.6 **	310.0 ± 22.5 **	2010.0 ± 4.5 **	540.0 ± 35.4
FI-TM	3220.0 ± 30.1 **	140.0 ± 8.1 **	2040.0 ± 5.6 *	460.0 ± 24.8 *
FI-TD	4740.0 ± 33.4 *	70.0 ± 5.1 **	1330.0 ± 8.5 **	360.0 ± 17.3 *

The values represent the mean ± standard deviation (SD), *n* = 3. FS-TM: Soluble fiber of *O. ficus indica* cladodes at an early maturity stage; FS-TD: Soluble fiber of *O. ficus indica* cladodes at a late maturity stage; FI-TM: Insoluble fiber of *O. ficus indica* cladodes at an early maturity stage; FI-TD: Insoluble fiber of *O. ficus indica* cladodes at a late maturity stage. * *(p* ≤ 0.05) compared to FS-TD; ** *(p* ≤ 0.05) compared to FS-TM.

**Table 3:** Weight gain, food intake, and dietary efficiency in rats fed with the experimental diets.

Parameters	Control	FS-TM	FS-TD	FI-TM	FI-TD
Initial weight (g)	131.0 ± 11.2	131.6 ± 10.7	132.0 ± 11.3	132.0 ± 10.4	127.2 ± 5.5
Final weight (g)	334.2 ± 28.7	360.4 ± 14.0	339.0 ± 29.0	368.2 ± 18.3	336.0 ± 19.4
Weight gain (g)	203.2 ± 14.2	228.8 ± 13.2	207.0 ± 11.3	236.2 ± 21.2	208.8 ± 11.6
Food intake (g)	1020.0 ± 48.7	1130.0 ± 54.1	1092.0 ± 87.4	1099.0 ± 89.9	1008.0 ± 97.4
Food efficiency ^†^	0.20 ± 0.01	0.20 ± 0.01	0.23 ± 0.02	0.22 ± 0.02	0.21 ± 0.01

The values represent the mean ± standard deviation (SD), *n* = 7. ^†^ Food efficiency: Weight gain (g)/food intake (g). FS-TM: Soluble fiber of *O. ficus indica* cladodes at an early maturity stage; FS-TD: Soluble fiber of *O. ficus indica* cladodes at a late maturity stage; FI-TM: Insoluble fiber of *O. ficus indica* cladodes at an early maturity stage; FI-TD: Insoluble fiber of *O. ficus indica* cladodes at a late maturity stage. No significant differences were observed between the values obtained from rats fed with the control diet and the rats fed the experimental diets (Tukey, *p* ≤ 0.05).

**Table 4:** Physical and mechanical properties of the femurs in rats fed with the experimental diets.

Parameters	Control	FS-TM	FS-TD	FI-TM	FI-TD
Length (cm)	3.6 ± 0.07	3.6 ± 0.07	3.6 ± 0.06	3.6 ± 0.03	3.6 ± 0.08
Weight (g)	1.05 ± 0.08	1.03 ± 0.04	1.14 ± 0.02	0.97 ± 0.05	0.90 ± 0.04 *
Width (mm)	4.3 ± 0.10	4.3 ± 0.09	4.4 ± 0.11	4.3 ± 0.15	3.9 ± 0.16 *
Thickness (mm)	3.1 ± 0.05	3.1 ± 0.06	3.2 ± 0.08 *	3.1 ± 0.05	3.0 ± 0.05
Compression test *F*max (N)	614.7 ± 10.6	671.4 ± 10.8 *	633.0 ± 13.8	587.4 ± 11.2	512.9 ± 13.1 *
Three point bending test *P*max (N)	87.8 ± 4.3	102.3 ± 1.9	109.7 ± 9.0 *	80.9 ± 6.8	63.7 ± 4.5 *
Young’s modulus E (N/mm^2^)	869.1 ± 21.9	1481.4 ± 50.9 *	2928.2 ± 49.4 *	1264.0 ± 53.9 *	830.1 ± 34.1

The values represent the mean ± SD *n* = 7. *F*_max_: Failure load evaluated by the compression test; *P*_max_: Failure load evaluated by the three-point bending test. FS-TM: Soluble fiber of *O. ficus indica* cladodes at early maturity stage; FS-TD: Soluble fiber of *O. ficus indica* cladodes at a late maturity stage; FI-TM: Insoluble fiber of *O. ficus indica* cladodes at an early maturity stage; FI-TD: Insoluble fiber of *O. ficus indica* cladodes at late maturity stage. * Significantly different from values of the bones from rats fed the control diet (*p* ≤ 0.05).

**Table 5:** Mineral content and Ca/P ratio within the femur of rats fed with experimental diets (mg/g).

Groups	Calcium	Phosphorus	Magnesium	Potassium	Ca/P ratio
Control	385.0 ± 5.7	92.8 ± 1.1	1.88 ± 0.01	0.378 ± 0.003	3.20 ± 0.009
FS-TM	360.6 ± 7.1 *	88.2 ± 0.6 *	1.73 ± 0.01 *	0.167 ± 0.001 *	3.17 ± 0.023
FS-TD	444.7 ± 2.7 *	91.4 ± 1.0	1.78 ± 0.01	0.229 ± 0.002 *	3.75 ± 0.183 *
FI-TM	242.5 ± 3.0 *	84.8 ± 0.7 *	1.63 ± 0.01 *	0.193 ± 0.001 *	2.20 ± 0.009 *
FI-TD	218.0 ± 3.5 *	88.4 ± 0.8 *	1.87 ± 0.10	0.106 ± 0.001 *	1.90 ± 0.013 *

The values represent the mean ± standard deviation (SD), *n* = 7. FS-TM: Soluble fiber of *O. ficus indica* cladodes at an early maturity stage; FS-TD: Soluble fiber of *O. ficus indica* cladodes at a late maturity stage; FI-TM: Insoluble fiber of *O. ficus indica* cladodes at an early maturity stage; FI-TD: Insoluble fiber of *O. ficus indica* cladodes at a late maturity stage. * Significantly different from the values of bones from rats fed with the control diet (*p* ≤ 0.05).

**Table 6:** Correlation coefficients between the BMD, mechanical, microstructural properties, and mineral content of the femurs in the experimental groups.

All Groups (n = 35)	BMD Childhood	BMD Puberty	BMD Adolescence	BMD Adulthood	Ca/P	Ct. Wi	Tb.Th	Tb.Sp
**Physical properties**								
Length	0.954 *	^†^	^†^	^†^	^†^	^†^	^†^	^†^
Weight	^†^	0.768	0.760 *	0.759 *	^†^	0.794 *	0.813 *	−0.766 *
Width	^†^	0.632 **	^†^	^†^	^†^	0.639 **		0.603 **
**Biomechanical properties**								
Fmax	^†^	0.787 **	0.709 *	0.822 *	^†^	0.819 *		−0.812 *
Pmax	^†^	0.916 *	0.877 *	0.845 *	^†^	0.838 *	0.704 *	−0.870 *
*E*	^†^	0.878 *	0.889 *	^†^	^†^	0.562 **	0.623 *	−0.654 *
**Microstructural properties**								
Ct.Wi	^†^	0.804 *	0.797 *	0.9789 *	0.956 *	^†^	0.861 *	−0.930 *
Tb.Th	^†^	^†^	0.736 *	0.845 *	0.905 *	^†^	^†^	−0.828 *
Tb.Sp	^†^	−0.858 *	−0.856 *	−0.933 *	−0.947 *	−0.931 *	−0.828 *	^†^
**Femur mineral content**								
Calcium	^†^	0.817 *	0.842 *	0.949 *	^†^	0.962 *	0.934 *	−0.937 *
Phosphorus	^†^	^†^	^†^	0.707 **	^†^	0.659 *	0.824 *	−0.593 **
Potassium	^†^	^†^	^†^	^†^	^†^	^†^	0.610 **	^†^
Magnesium	^†^	^†^	^†^	^†^	^†^	^†^	^†^	^†^
Ca/P ratio	^†^	^†^	0.856 *	0.946 *	^†^	^†^	^†^	^†^

*F*_max_: Failure load evaluated by the compression test; *P*_max_: Failure load evaluated by the three-point bending test; *E*: Young’s modulus; Ct.Wi: Cortical width; Tb.Th: Trabecular thickness; Tb.Sp: Trabecular separation; * *p* < 0.001; ** *p* < 0.01; ^†^ indicates a correlation coefficient < 0.5; *p*: Significance level.
